# Validation of *Aedes aegypti* Aag-2 cells as a model for insect immune studies

**DOI:** 10.1186/1756-3305-5-148

**Published:** 2012-07-24

**Authors:** Ana Beatriz Ferreira Barletta, Maria Clara L Nascimento Silva, Marcos H Ferreira Sorgine

**Affiliations:** 1 Laboratório de Bioquímica de Artrópodes Hematófagos, Instituto de Bioquímica Médica, Programa de Biologia Molecular e Biotecnologia, Universidade Federal do Rio de Janeiro, Rio de Janeiro, Brazil; 2 Instituto Nacional de Ciência e Tecnologia em Entomologia Molecular (INCT-EM), Rio de Janeiro, Brazil; 3 Trifermed, ESADECREAPOLIS innovation lab, Barcelona, Spain

## Abstract

**Background:**

The understanding of mosquito immune responses can provide valuable tools for development of novel mosquito control strategies. Aiming the study at insect innate immunity, continuous insect cell lines have been established and used as research tools due to the fact that they constitute more homogeneous, sensitive, and reproducible systems than the insects from which they originated. More recently, Aag-2, an *Aedes aegypti* cell lineage, began to be frequently used as a model for studies of mosquito immunity. Nevertheless, to our knowledge, no study has systematically characterized the responses of Aag-2 cell line against different kinds of pathogens and compared its response to those exhibited by whole mosquitoes. For this reason, in this study we characterized gene expression profiles of the Aag-2 cell line in response to different kinds of immune challenges, such as Gram negative and positive bacteria, fungi and viruses, comparing the obtained results with the ones already described in the literature for whole mosquitoes.

**Methods:**

*Aedes aegypti* Aag-2 cells were exposed to different immune stimuli (gram-positive and gram negative heat inactivated bacteria, zymosan or Sindbis virus) for 24 hours and the expression of selected marker genes from toll, IMD and Jak/STAT pathways was analyzed by qPCR. Also, cells were incubated with fluorescent latex beads for evaluation of its phagocytosis capacity.

**Results:**

Aag-2 cells were stimulated with two concentrations of heat-killed Gram negative (*Enterobacter cloacae*) or Gram positive (*Micrococcus luteus*) bacteria, Zymosan or infected with Sindbis virus and the expression of key genes from the main immune related pathways, Toll, IMD and Jak/STAT, were investigated. Our results suggest that Toll and IMD pathways are activated in response to both Gram positive and negative bacteria and Zymosan in Aag-2 cells, displaying an immune profile similar to those described in the literature for whole mosquitoes. The same stimuli were also capable of activating Jak/STAT pathway in Aag-2 cells. Infection with Sindbis virus led to an up-regulation of the transcription factor STAT but was not able to induce the expression of any other gene from any of the pathways assayed. We also showed that this cell line is able to phagocytose latex beads in culture.

**Conclusions:**

Our results characterize the expression profile of Aag-2 cells in response to different immune stimuli and demonstrate that this cell lineage is immune-competent and closely resembles the response described for whole *Ae. aegypti* mosquitoes. Hence, our findings support the use of Aag-2 as a tool to comprehend *Ae. aegypti* immune response both at cellular and humoral levels.

## Background

*Aedes aegypti* mosquitoes are important vectors of viral diseases, such as Yellow and Dengue fever, which have significant impact on human morbidity and mortality. The incidence of Dengue has grown around the world, mainly in the tropics and subtropics, in the last decades. Nowadays, about 2.5 billion people are currently at risk of infection [1]. Traditional strategies currently employed for the control of the disease vector, *Ae. aegypti*, such as insecticide applications are becoming more and more ineffective due to the rapid development of resistance by this vector [2]. This situation reinforces the need to understand the biology of the vector/pathogen interaction. One aspect that plays an important role in the interaction between a pathogen and its host is immunity. Insect innate immune responses are controlled by three major signaling pathways, the Toll, the Immune Deficiency (IMD) and the Janus kinase (Jak) – signal transducer and activator of transcription (STAT) pathways [3-7].

The Toll pathway is activated mainly by Gram-positive bacteria, fungi [8,9] and viruses [10], and largely controls the expression of antimicrobial peptides (AMPs). When pathogen-associated molecular patterns (PAMPs) are recognized, a signaling cascade is activated and NF-ĸB factor(s) is/are translocated to the nucleus to initiate the transcription of molecules involved with the immune response [11]. In *Drosophila melanogaster,* DIF and Dorsal are NF-ĸB homologues factors involved in the activation of the Toll pathway, although in mosquitoes only one Toll related NF-ĸB factor is present, REL 1, which is homologous to dorsal.

Upon activation of IMD pathway, mainly by Gram negative bacteria in *D. melanogaster*, another NF-ĸB factor, Relish, is translocated to the nucleus leading to the transcription of the pathway effector molecules [12,13].

Since the sequencing of the *Ae. aegypti* genome in 2007 [14] putative orthologs of *D. melanogaster* and *An. gambiae* immune genes have been identified and there has been an important growth of the knowledge about how this mosquito is able to fight against different pathogens such as viruses, bacteria and worms [15].

Continuous insect cell lines have been an important research tool for insect biologists since Grace developed the first reported insect cell line from a moth [16]. Cell lineages constitute homogeneous, sensitive and reproducible systems, allowing the detection of very subtle changes in the response to different kinds of pathogens and stimuli. For these reasons, cells from *D. melanogaster* (especially S2 cells), *An. gambiae* (5.1* and Sua5B), *Lutzomya longipalpis* (LL5 cells), *Ae. albopictus* (especially C6/36 cells), among other insects, have long been used to investigate different aspects of insect immunity, being essential tools for the construction of the knowledge we have today regarding Toll, IMD, Jak/STAT and RNAi pathways in insects [17-24].

Most of the studies that have focused on mosquito immune responses against pathogens have employed the lineage C6/36 [17,25-27], a lineage established from *Ae. albopictus* larvae homogenates by Singh [28]. C6/36 has been used to understand the regulation of the synthesis and secretion of several important proteins/processes, such as defensin [29], cecropin [24] or phagocytosis [19] but mostly this cell has been used to study aspects of insect-virus relations [17]. This is because one of the main features of C6/36 is the capacity of growing a very broad spectrum of viruses and producing higher virus titers than any other cell [30]. It is now known that this property is, at least partially, due to the lack of a functional RNAi response in these cell lineages [30].

Recently, Aag-2, an *Ae. aegypti* cell lineage of embryonic origin [31] began to be used as a model for studies of mosquito immunity [32-35]. Nevertheless, to our knowledge, no work has been published systematically characterizing the responses of the Aag-2 cell line, against different kinds of pathogens and comparison of the response to the one exhibited by whole mosquitoes, in this way “validating” this cell line as a tool for immune investigations. For this reason, in this study we characterized the gene expression profile of the Aag-2 *Ae. aegypti* cell line in response to different kinds of immune challenges, such as Gram negative and positive bacteria, fungi and viruses. Our results show that the Aag-2 lineage is immunocompetent and also that the immune responses elicited closely resemble the responses described for whole *Ae. aegypti* mosquitoes.

## Methods

### Cell Culture

*Aedes aegypti* Aag-2 cells were maintained at 28 °C in Schneider´s *Drosophila* medium with L-glutamine (Gibco) supplemented with 10% Fetal Bovine Serum (FBS) (Cultilab) and were passaged at a 1:10 dilution every 4–5 days. For this passage, cells were released from the culture flask with 0.25% trypsin solution (Gibco). Once in solution, cells were transferred to well plates for the indicated treatments or to another culture flask for maintenance.

### Treatments and Viral Infection

Cells were seeded in a 12-well plate (TPP- Tecno Plastic Products) at 80-90% of confluence, approximately 2x10^5^ cells per well and incubated overnight for adherence to occur. For bacterial challenge, cells were incubated with two different heat killed bacteria as previously described [34]: *Micrococcus luteus*, a Gram positive bacteria and *Enterobacter cloacae*, a Gram negative bacteria. Aag-2 cells were incubated with 100 bacteria per cell (10^7^ bacteria/ well) or 1000 bacteria per cell (10^8^ bacteria/ well). Cells were also stimulated with Zymosan A (Sigma-Aldrich), a yeast cell wall sugar that consists of protein-carbohydrate complexes. Two different concentrations of Zymosan A (0.5 mg/mL and 1 mg/mL) were used in the assays as previously described [36]. Before use, Zymosan A suspension was autoclaved to, eventually, eliminate any contaminant live yeast. The incubation with both bacteria and Zymosan A lasted 24 hours. For viral infection, cells were infected with Sindbis virus using a MOI (Multiplicity of Infection) of 10, as previously described [37]. Cells were incubated in the presence or absence of virus in Schneider´s *Drosophila* medium with L-glutamine (Gibco) without FBS for 1 hour at 37°C. After that, cells were washed twice with phosphate-buffered saline (PBS) and medium supplemented with 10% SFB was replaced in all wells. The incubation lasted 24 hours and the cells were maintained at 28°C. Each figure represents at least five biological replicates.

### RNA Extraction, cDNA synthesis and Quantitative PCR

Total RNA from cells in all conditions was extracted using the TRIZOL reagent (Invitrogen) following the manufacturer's instructions. RNA was treated with DNAse I (Fermentas) and first-strand cDNA synthesis was carried out using High-Capacity cDNA Reverse transcription kit (Applied Biosystems). The efficiency of the experimental set for each gene was tested with serial dilutions of cDNA and was only used if the resultant efficiency was ≥ 90%. Each PCR reaction (15 μL volume) contained diluted cDNA, 2 × Power SYBR Green Master Mix (Applied Biosystems) and 300 nM of forward and reverse primers. Quantitative PCR was performed in a 7500 Real Time PCR System (Applied Biosystems) using Applied Biosystems recommended qPCR conditions (20 seconds at 95°C followed by 40 cycles of 95°C for 1 second and 20 seconds at 60°C followed by a melting curve to assure a simple product was amplified). The comparative ΔΔCt method was used to evaluate changes in gene expression levels and all standard errors were calculated based on ΔCt as described in Applied Biosystems User Bulletin #2 (http://www3.appliedbiosystems.com/cms/groups/mcb_support/documents/generaldocuments/cms_040980.pdf). The *Ae. aegypti* ribosomal protein 49 gene RP-49 was used as endogenous control (accession number AAT45939), based on previous data [38]. Primer sequences used are described on Table 1. Each figure represents at least five biological replicates with three technical replicates for each sample.

**Table 1:** Primers used in this study

**Accession number**	**Gene**	**Primer sequence**	**Melting temperature (°C)**	**R**^**2**^**efficiency Curve**	**Primer efficiency**
AAEL007696	aaREL 1 For	**GACTCGTCGGAGCTGAAATC**	81.1	0.9989	1.1516
	aaREL 1 Rev	**CGGTTTGTTCAGGTTGTTGA**			
AAEL007624	aaREL 2 For	**TCTGTCGGCAGATGAAGTGA**	79.7	0.9999	1.1144
	aaREL 2 Rev	**GCACTGGAATGGAGAATCAAA**			
AAEL000709	Cactus For	**TCTTGCGTTGAAGTGAGTGG**	79.2	0.993	1.034
	Cactus Rev	**GACCCTCTGAAAGGGAAAGG**			
AAEL003841	Defensin For	**GATTCGGCGTTGGTGATAGT**	81.9	0.9992	0.9955
	Defensin Rev	**TTATTCAATTCCGGCAGACG**			
AAEL010083	IMD For	**TCGTCAAACTCGGTTTTCCT**	78.9	0.9925	1.1068
	IMD Rev	**TGGCGGAGTTGAAGGTAAAG**			
AAEL007768	Myd88 For	**CGATGCGTTCATTTTGTTTG**	76.8	0.9889	1.03
	Myd88 Rev	**CACCGCTCAGAAATCAGCTT**			
AAT45939	RP49 For	**GCTATGACAAGCTTGCCCCCA**	83.7	0.9879	1.2002
	RP49 Rev	**TCATCAGCACCTCCAGCT**			
AAEL007765	Serpin For	**ACGTGATGGATTGGATGGAG**	79.2	0.9995	1.0149
	Serpin Rev	**GTGCCTGCACTTGTTTCTGA**			
AAEL009692	STAT For	**CACACAAAAAGGACGAAGCA**	75.7	1	1.1797
	STAT Rev	**TCCAGTTCCCCTAAAGCTCA**			
AAEL001794	TEP For	**ATTTTTGACGGCTTTTGTGG**	78.9	0.9992	1,0604
	TEP Rev	**TGGATTACTTGCCCCACTTC**			

### Phagocytosis assay

Cells were seeded in a 24-well plate with a glass slide at 60% of confluence. The phagocytosis assay was performed as in Mizutani *et al*. (2003) [19]. The medium was removed and cells were incubated with 4 x 10^7^/mL 1.0 μm fluorescent red latex beads carboxylate-modified polystyrene (Sigma Aldrich) per well in Mg^2+^-free Hank`s solution (Sigma Aldrich) and incubated for 1 hour at 28°C or 4°C (control). After this period, cells were washed with phosphate-buffered saline (PBS) and then fixed with 3.7% formaldehyde for 30 minutes. After fixation, 0.4% Trypan blue solution (Sigma Aldrich) in phosphate-buffered saline (PBS) was added to quench the fluorescence from the extracellular beads. Cells were observed in a fluorescence microscope Zeiss Axioskop 40 with an Axiocam MRC5 using a Zeiss-15 filter set (excitation BP 546–612; beam splitter FT 580; emission LP 590). Differential interference contrast (DIC) images were acquired with a Zeiss AxioObserver, which was also used for some fluorescence images, with Zeiss-15 filter set (excitation BP 546–612; beam splitter FT 580; emission LP 590) for fluorescent red latex beads. Comparison of fluorescence levels among distinct images was performed under identical conditions, using the same objectives, microscopes and similar exposure times in each experiment.

### Statistical analysis

Quantitative PCRs were statistically analyzed by ANOVA followed by Dunnett`s multiple comparison test. Analyzes were performed on ΔCt data, before normalization, using GraphPad Prism statistical software package (Prism 5.01; GraphPad Software, Inc., San Diego, CA). Asterisks indicate significant differences (*p < 0.05; **p < 0.01; ***p < 0.001).

## Results

Cells were stimulated with two concentrations of heat-killed Gram negative (*Enterobacter cloacae*) and Gram positive (*Micrococcus luteus*) bacteria and with Zymosan A, a glucan from yeast cell walls, or infected with Sindbis virus.

### Toll pathway

**Figure 1:**
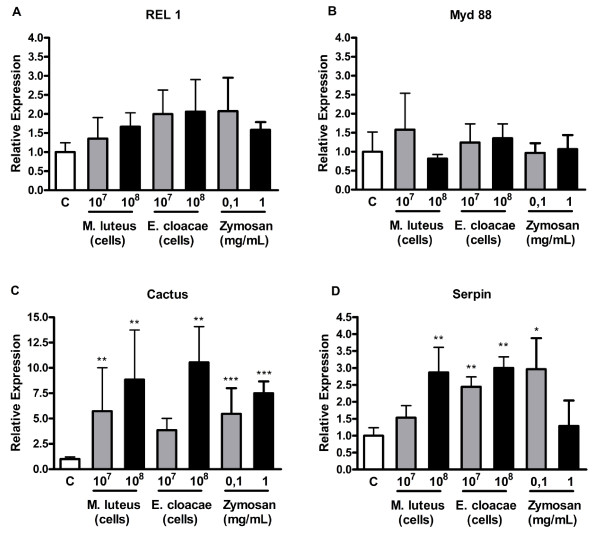
**Expression of Toll pathway marker genes after exposure of*Aedes aegypti*Aag-2 cells to Gram-positive (*M. luteus*) or negative (*E. cloacae*) bacteria or to Zymosan.** Cells were incubated with the indicated amounts of the indicated stimuli for 24 hours and the expression of REL 1(**A**), MyD88 (**B**), cactus (**C**) or serpin (**D**) was analyzed by qPCR using RP-49 gene as endogenous control. Each bar represents the relative expression and standard error of the analyzed genes, calculated as described in Methods section.

When cells were exposed to either Gram positive or Gram negative bacteria or to Zymosan, the genes from the Toll pathway presented a bimodal expression profile. The transcription factor REL 1 (Figure 1A) and the adaptor protein Myd88 (Figure 1B) did not change expression in comparison to control cells after any of the treatments, although a trend towards increase can be observed for REL 1 upon incubation of the cells with all immune elicitors.

The homolog of the NF-ĸB inhibitor (IĸB), cactus, on the other hand, was significantly induced when cells were incubated with Zymosan (Figure 1C). The same profile was observed when cells were stimulated with the Gram positive bacteria or with high amounts of Gram negative bacteria (Figure 1C). An effector molecule of the Toll pathway, a serine protease inhibitor, serpin 27A, was also overexpressed in response to the two types of bacteria in both concentrations (Figure 1D). However, in cells incubated with Zymosan, the levels of serpin 27A mRNA were elevated only upon incubation of the cell with the smallest amount (Figure 1D).

**Figure 2:**
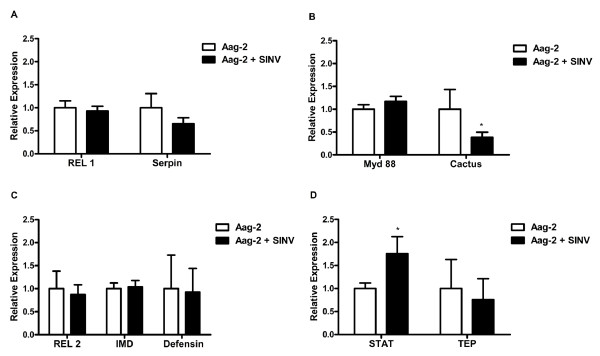
**Expression of Toll, IMD and Jak/STAT pathway marker genes after exposure of*Aedes aegypti*Aag-2 cells Sindbis virus.** Cells were infected with Sindbis virus using a MOI of 10. After 24 hour of incubation at 28°C, the expression marker genes from the Toll (**A**,**B**), IMD(**C**) or Jak/STAT (**D**) pathways was analyzed by qPCR using RP49 gene as endogenous control. Each bar represents the relative expression and standard error of the analyzed genes, calculated as described in Methods section.

When cells were infected with the alphavirus Sindbis, any significant increase in the expression profile of the analyzed Toll pathway related genes was observed. In fact, cactus and serpin presented a decrease in mRNA levels upon infection of the cells (Figure 2A and 2B).

### IMD Pathway

**Figure 3:**
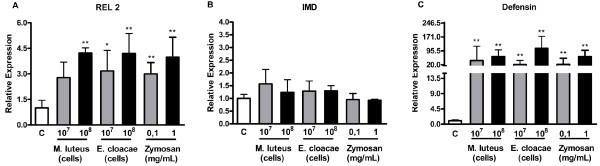
**Expression of IMD pathway marker genes after exposure of*Aedes aegypti*Aag-2 cells to Gram-positive (*M. luteus*) or negative (*E. cloacae*) bacteria or to Zymosan.** Cells were incubated with the indicated amounts of the indicated stimuli for 24 hours and the expression of REL 2 (**A**), IMD (**B**) or defensin (**C**) was analyzed by qPCR using RP49 gene as endogenous control. Each bar represents the relative expression and standard error of the analyzed genes, calculated as described in Methods section.

REL 2, the NF-ĸB homologue transcription factor of IMD pathway, showed a 3 to 5 fold expression increase in response to incubations of Aag-2 cells with all concentrations of Zymosan, Gram negative and Gram positive bacteria (Figure 3A). The adaptor protein IMD, showed the same pattern observed for Myd88, the adaptor protein of the Toll pathway, having its expression unaltered in response to all stimuli (Figure 3B). One of the IMD pathway effectors, defensin was highly up-regulated in response to both Gram positive or negative bacteria and Zymosan (Figure 3C). Interestingly, the level of defensin up-regulation was higher than any other molecule analyzed.

The IMD pathway showed any change in gene expression in cells infected with Sindbis virus (Figure 2C).

### Jak/STAT Pathway

**Figure 4:**
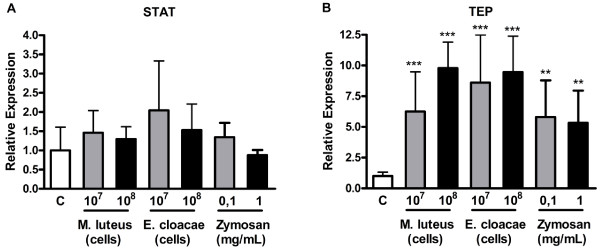
**Expression of Jak/STAT pathway marker genes after exposure of*Aedes aegypti*Aag-2 cells to Gram-positive (*M. luteus*) or negative (*E. cloacae*) bacteria or to Zymosan.** Cells were incubated with the indicated amounts of the indicated stimuli for 24 hours and the expression of STAT (**A**) or TEP (**B**) was analyzed by qPCR using RP49 gene as endogenous control. Each bar represents the relative expression and standard error of the analyzed genes, calculated as described in Methods section.

In the Jak/STAT pathway, transcription factor STAT did not show significant changes in response to Gram positive or negative bacteria or Zymosan at any concentration tested (Figure 4A). A thiol-ester motif-containing *protein*, TEP [39] was up-regulated in response to Gram negative and Gram positive bacteria (Figure 4B).

When the cells were infected with Sindbis virus, the transcription factor STAT was the only molecule analyzed that was significantly up-regulated (Figure 2D). However, the expression of TEP showed no changes in infected cells (Figure 2D).

### Phagocytosis

**Figure 5:**
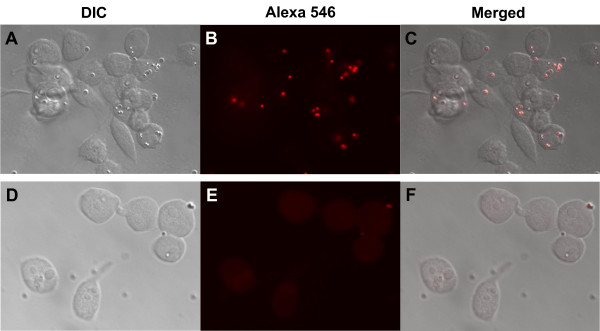
***Aedes aegypti*Aag-2 cells are able to phagocytose.** Aag-2 cells were incubated with 2 × 10^7^ 1.0 μm fluorescent red latex beads (Sigma Aldrich) for 1 hour at 28°C (A-C) or 4°C (D-F, control). After this period, cells were fixed with formaldehyde 3.7% for 30 minutes and 0.4% Trypan blue solution in phosphate-buffered saline (PBS) and observed under a fluorescence microscope. (**A** and **D**) Differential interference contrast (DIC) images. (**B** and **E**) Fluorescence image. (**C** and **F**) Merge. Magnification 100X.

An important characteristic of immune competent cells is the capacity to phagocytose foreign bodies such as bacteria or fungi. To evaluate if the cell line Aag-2, besides presenting a similar expression profile to immune cells, like hemocytes, was capable of phagocytosing microorganisms, cells were maintained in culture, incubated with fluorescent latex beads for 1 hour and observed in an epifluorescence microscope. Latex beads could be observed in the cytoplasm of virtually all cells in the well (Figure 5A-C), confirming the ability of this cell to phagocytose. As control, cells were incubated under the same conditions at 4°C to inhibit phagocytosis (Figure 5D-F). Incubations with Trypan Blue were made after the fixation step to quench the fluorescence from extracellular beads.

## Discussion

Recently, insect cell lines have proven to be highly useful for such studies, since they are easy to handle and grow and typically produce homogeneous and reliable results.

In 1968, Peleg developed an *Ae. aegypti* embryonic cell line [31], Aag-2. Curiously for almost 30 years this lineage was neglected until Gao *et al*. (1999) [34] published a paper describing the secretion of a defensin by this cell in response to heat-killed Gram positive bacteria. After that, several groups started using Aag-2 cells and it has now become clear that this lineage has several advantages over C6/36 as a model cell for the genus *Aedes*. One important point is that, unlike *Ae. albopictusAe. aegypti* has a sequenced genome [14]. This is an extremely important characteristic of this cell since having a genome makes it tremendously easier to identify genes of interest and use conventional methodologies to study gene expression, such as qPCR, but also allows the use of high-throughput research methodologies, such as microarrays [32]. Unlike C6/36, Aag-2 has a functional RNAi pathway [40]. This fact not only makes this cell a more accurate model to study viral infection but also allows the use of reversal genetic strategies, especially RNAi based approaches in this cell.

When the cells were exposed to Gram positive or negative bacteria or Zymosan, expression of cactus and serpin 27A were significantly increased. These results are consistent with an activation of the Toll pathway, since the expression of cactus is increased upon Toll pathway activation [41], and in whole mosquitoes, the expression of serpin 27A is responsive to the Toll pathway and is totally abolished upon knock down of REL 1 [42]. Interestingly, we also observed an activation of this pathway when the cells were incubated with Gram negative bacteria (*E. cloacae*), as seen in whole mosquitoes challenged with the same bacterium [35]. This finding reinforces the idea that Aag-2 immune responses are very similar to the mosquito.

With the exception of the adapter protein IMD, all other IMD related genes investigated were induced by the three stimuli used. In *D. melanogaster*, defensin expression is controlled mainly by the Toll pathway, but in *Ae. aegypti*, this gene is controlled by the IMD pathway [43,44]. Defensin was induced more than 100 fold upon incubation with *E. cloacae*.

These results are significantly different for *Drosophila*, where the IMD pathway is activated only for Gram negative bacteria and not Gram positive or fungi [45]. Nevertheless, the immune pattern exhibited by Aag-2 cell accords to the pattern observed for whole mosquitoes. In mosquitoes, Gram-positive bacteria are able to activate the IMD pathway and the peptides produced by this pathway, defensin among them, are able to efficiently impair the growth of Gram-positive bacteria and increase mosquito survival upon infection [44,46].

From the “classical” mosquito immune pathways, Jak/STAT is the pathway about which less is known. The *Aedes* Jak/STAT pathway can be activated by fungal and viral infections [39,47]. Here, we analyze two genes from this pathway, the transcription factor STAT and TEP, a gene shown to be under control of the Jak/STAT pathway [39]. STAT expression was not altered when incubated with bacteria or Zymosan. This is not surprising since the increase of STAT protein levels is not necessarily required for the activation of the pathway. TEP expression significantly increased in response to all these challenges, suggesting that all of them are able to activate Jak/STAT pathway. Again, this is in accordance with the data available in the literature for mosquitoes. *Beauveria bassiana*, an entomopathogenic, fungus activates Jak/STAT pathway [47]. Bartholomay *et al*. (2007) [48] showed that hemocytes of mosquitoes infected with a Gram negative (*E. coli*) or Gram positive (*M. luteus*) bacteria present significant increases in TEP expression.

When Aag-2 cells were infected with Sindbis virus, from all the genes of the three pathways we assayed, only STAT was significantly up-regulated. Although the mechanisms employed by mosquitoes to fight against flavivirus, such as dengue, have recently been revealed, with Toll and Jak/STAT, but not IMD, playing important roles in the control of virus titers [10,39], little is known about how mosquitoes control alphavirus infections. Sanders *et al*. (2005) [49] in a microarray study of *Aedes* infected with Sindbis virus, identified a transient increase in REL 1 expression only in the first day after infection. Curiously, no other gene from the Toll pathway was up-regulated in the array. On the other hand, depending on the Sindbis or *Aedes* strains used in this assay, no gene from the Toll pathway could be up-regulated in Sanders *et al.* (2005) [49] assay (Gill S, personal communication). In fact, we show that when Aag-2 cells were infected with Sindbis virus, no genes from the Toll pathway were up-regulated. On the contrary, one gene from the Toll pathway, cactus, was actually down-regulated.

The observed up-regulation of STAT upon Sindbis infection, could be related with the known involvement of this pathway in the control of viral infections [4,10]. Unfortunately, the lack of papers describing immune aspects, other than RNAi, of *Aedes*-Sindbis interaction makes it difficult to understand if the increased expression of STAT exhibited by Sindbis infected Aag-2 is also a characteristic of infected mosquitoes. Also noteworthy is the fact that, although STAT is up-regulated, there is no increase in TEP expression. This probably points to the fact that, upon Sindbis infection, only a subset of Jak/STAT regulated genes is activated.

Recent studies have unequivocally shown that a phagocytic response is as important as the humoral one for insects to fight pathogens [50]. Besides, the entrance of many viruses such as Dengue into host cell is dependent on active phagocytosis [51]. Our results show that, like mosquito hemocytes and other insect cultured cells, when exposed to latex beads Aag-2 efficiently phagocytes these bodies, revealing one more characteristic shared with other insect immunocompetent cells.

## Conclusions

After examining Aag-2 cells immune responses against Gram-positive and negative bacteria, fungi and Sindbis virus, besides its capacity to phagocyte strange bodies, it is our conclusion that this cell lineage responds to these stimuli in a very similar way to that described for whole mosquitoes and constitutes a good model for insect immune studies.

## Competing interests

The authors declare that they have no competing interests.

## Authors’ contributions

Conceived and designed the experiments: ABBF MCLNS MHFS. Performed the experiments: ABBF. Analyzed the data: ABBF MCLNS MHFS. Contributed reagents/materials/analysis tools: MHFS. Wrote the paper: ABBF MHFS. All reviewers read and approved the final version of the manuscript.
